# Characterization of antibiotic overuse for common infectious disease states at hospital discharge

**DOI:** 10.1017/ash.2023.497

**Published:** 2023-12-12

**Authors:** Rebecca Zodrow, Andrew Olson, Stephanie Willis, Dennis Grauer, Megan Klatt

**Affiliations:** 1 Department of Pharmacy, The University of Kansas Health System, Kansas City, KS, USA; 2 School of Pharmacy, The University of Kansas, Lawrence, KS, USA

## Abstract

**Objective:**

To evaluate rates of and outcomes associated with antibiotic overuse at hospital discharge for patients with common infectious diseases states.

**Design:**

Single-center, respective cohort study.

**Setting:**

A large, academic medical center in the Midwest United States.

**Patients:**

Adult patients who received antibiotics for community-acquired pneumonia (CAP), uncomplicated cystitis, or mild, non-purulent cellulitis. Patients were excluded if they did not receive antibiotic(s) upon hospital discharge, were pregnant, severely immunocompromised, had concomitant infections, died during hospitalization, or were transferred to another hospital or to an intensive care unit.

**Methods:**

Data were abstracted from the electronic medical record of ambulatory antibiotic orders for included patients based on inpatient encounters from August 1, 2021 through July 31, 2022.

**Results:**

Of the 182 patients included in the study, antibiotic overuse was common for all three infectious disease states: CAP (*n* = 87/125, 69.6%), uncomplicated cystitis (*n* = 21/28, 75.0%), mild, non-purulent cellulitis (*n* = 28/29, 96.6%). The prevailing reason for overuse was excessive antibiotic duration (*n* = 127/182, 69.8%; mean antibiotic duration 5.39 vs. 8.32 days, *p* = 0.001). Antibiotic overuse was associated with approximately one additional day in the hospital (2.48 vs. 3.32 days, *p* = 0.001), and an increase in emergency department visits within 30 days after discharge (1 vs. 31, *p* = 0.001) compared to patients without antibiotic overuse at discharge.

**Conclusion:**

Antibiotic overuse was prevalent upon hospital discharge for these three common infectious disease states. Transitions of care should be prioritized as an area for antimicrobial stewardship intervention.

## Introduction

Antimicrobial stewardship efforts ensure the safe and judicious use of antimicrobials. Hospital antimicrobial stewardship programs have historically focused on optimizing the use of antibiotics during inpatient stays.^1^ However, recent publications have demonstrated inappropriate antimicrobial prescribing at hospital discharge and the need for innovative stewardship strategies for this space.^2,3^

Approximately 1 in 8 patients discharged from an inpatient setting receive an antimicrobial at discharge.^4^ In their multi-hospital study, Vaughn and colleagues evaluated two infectious conditions and reported nearly half of patients experienced antibiotic overuse at discharge where overuse was characterized by unnecessary antibiotic use, excessive duration, and suboptimal use of fluoroquinolone therapy. Importantly, antibiotic overuse varied significantly between sites (15.9%–80.6%) and was less commonly observed in academic hospitals.^2^ Our study aimed to characterize discharge antibiotic prescribing for three common infectious disease states, building on the work by Vaughn and colleagues, at a large, academic medical center to determine the extent of antimicrobial overuse and opportunities for antimicrobial stewardship intervention.

## Methods

### Study design and setting

This single-center, retrospective cohort study included adult patients age 18 and older admitted to The University of Kansas Health System and prescribed antibiotic(s) at discharge for community-acquired pneumonia (CAP), uncomplicated cystitis, or mild, non-purulent cellulitis between August 1, 2021 and July 31, 2022. Patients were identified for inclusion based on antibiotic indication via review of admission diagnostic codes and chart review confirmation. The following patient populations were excluded: patients who did not receive antibiotic(s) upon hospital discharge, died during hospitalization, and transferred to another hospital or an intensive care unit, and patients for whom standard regimens may be inappropriate such as pregnant patients, severely immunocompromised, and patients with concomitant infections. This study was approved by the Institutional Review Board of the University of Kansas Medical Center.

### Clinical outcomes

The primary outcome was percentage of patients with antibiotic overuse at hospital discharge, defined as meeting at least one of the following: unnecessary antibiotic use, excessive antibiotic duration, and suboptimal fluoroquinolone use. Secondary outcomes included mortality, readmission, emergency department (ED) visit, incidence of *Clostridioides difficile* infection, and provider-documented or patient-reported antibiotic-associated adverse effect(s) all assessed within 30 days of hospital discharge.

### Data analysis

Descriptive statistics were reported as median and interquartile range or mean and standard deviation based on normality of data, where applicable. Quantitative variables were compared using 2-sided chi-squared, student t-test, or analysis of variance as appropriate (ANOVA), with a *p*-value of <0.05 considered statistically significant.

## Results

In total, 2,865 ambulatory antibiotic prescriptions were evaluated of which 182 patients met inclusion criteria. Antibiotic overuse was common for all three disease states: CAP (*n* = 87/125, 69.6%), uncomplicated cystitis (*n* = 21/28, 75.0%), and mild, non-purulent cellulitis (*n* = 28/29, 96.6%). Excessive antibiotic duration occurred most frequently at 65.6%, 60.7%, and 96.6% of patients diagnosed with CAP, uncomplicated cystitis, and mild, non-purulent cellulitis, respectively. When comparing patients who experienced antibiotic overuse to those who did not, overuse was associated with approximately one more inpatient hospital day (2.48 vs. 3.32 days, *p* = 0.001) and, on average, three extra days of antibiotics (5.39 vs. 8.32 days, *p* = 0.001) (Table 1). Regarding secondary outcomes, there was a statistically significant increase in the amount of ED visits within 30 days (2.2% vs. 22.8%, *p* = 0.001) while all other secondary outcomes findings were non-significant.

**Table 1.** Characteristics of patients with and without antibiotic overuse at hospital discharge for all disease states

	No antibiotic overuse at discharge (*n* = 46)	Antibiotic overuse at discharge (*n* = 136)	*P* value
Race – White, *n* (%)	30 (65.2)	100 (73.5)	0.281
Sex – Female, *n* (%)	24 (52.2)	77 (56.6)	0.600
Age, mean (SD)	64.4 (16.5)	63.8 (16.4)	0.829
Charlson Comorbidity Index, mean (SD)	3.93 (2.56)	3.65 (2.33)	0.481
Length of stay, mean (SD)	2.48 (1.01)	3.32 (1.70)	0.001
Infectious disease state, *n* (%)			0.011
Community-acquired pneumonia	38 (82.6)	87 (64.0)	
Uncomplicated urinary tract infection	7 (15.2)	21 (15.4)	
Mild, non-purulent cellulitis	1 (2.2)	28 (20.6)	
Antibiotics days, mean (SD)			
Antibiotic duration, days	5.39 (0.80)	8.32 (2.73)	0.001
Antibiotic overuse after discharge, days	0 (0)	3.47 (2.61)	N/A

## Discussion

In this study of 182 patients, nearly 3 out of every 4 patients prescribed an antibiotic at hospital discharge for CAP, uncomplicated cystitis, and mild, non-purulent cellulitis met criteria for antibiotic overuse. Excessive antibiotic duration was the most common type of antibiotic overuse for all three infectious conditions resulting in an average of three additional days of antibiotics. Given the high rates of antibiotic overuse at hospital discharge at our institution (74.7% overall), implementation of novel approaches to antimicrobial stewardship at transitions of care is crucial to improving antibiotic use. In the present state, inpatient pharmacists at our health system review discharge medications for most patients prior to discharge, however, no formal interventions have been developed. The Reducing Overuse of Antibiotics at Discharge Home Framework provides a three-tiered system for developing antimicrobial stewardship strategies to reduce antibiotic overuse at hospital discharge.^3^ In their study of 800 patients receiving antibiotics at discharge, Mercuro and colleagues demonstrated a significant improvement in optimal antibiotic prescribing with targeted, pharmacist-led interventions.^5^ Thus, creation of a pharmacist-led intervention at transitions of care may be an effective means by which to reduce antibiotic overuse within ours and other institutions.

Our study benefits from several strengths. First, the definitions for each disease state and the categories for overuse are highly specific and supported by practice guidelines. Second, a thorough patient investigation and chart review allowed for immunocompromised patients, which might skew outcomes, to be confidently excluded. However, several limitations exist. First, our study had a small sample size, as many patients were excluded due to inaccurate antimicrobial indications, resulting in less than 30 patients included in the uncomplicated cystitis and mild, non-purulent cellulitis arms. Second, the observational study design is susceptible to confounding. Lastly, the study had a low incidence of secondary outcome events, and low rates of such outcomes may be due to a high degree of patients lost to follow-up.

In summary, our analysis demonstrated a high amount of antibiotic overuse at hospital discharge for patients treated for three common infectious disease states at a large, academic medical center. Future antimicrobial stewardship efforts in this space should be tailored based on institution-specific data with enhanced pharmacist involvement to ensure optimized antimicrobial use.

## References

[1] Barlam TF , Cosgrove SE , Abbo LM , et al. Implementing an antibiotic stewardship program: Guidelines by the Infectious Diseases Society of America and the Society for Healthcare Epidemiology of America. Clin Infect Dis 2016;62:e51–e77. doi: 10.1093/cid/ciw118 27080992 PMC5006285

[2] Vaughn VM , Gandhi TN , Chopra V , et al. Antibiotic overuse after hospital discharge: A multi-hospital cohort study. Clin Infect Dis 2021;73:e4499–e4506. doi: 10.1093/cid/ciaa1372 32918077 PMC7947015

[3] Vaughn VM , Hersh AL , Spivak ES. Antibiotic overuse and stewardship at hospital discharge: The reducing overuse of antibiotics at discharge home framework. Clin Infect Dis 2022;74:1696–1702. doi: 10.1093/cid/ciab842 34554249 PMC9070833

[4] Dyer AP , Dodds Ashley E , Anderson DJ , et al. Total duration of antimicrobial therapy resulting from inpatient hospitalization. Infect Control Hosp Epidemiol 2019;40:847–54. 31134880 10.1017/ice.2019.118

[5] Mercuro NJ , Medler CJ , Kenney RM , et al. Pharmacist-driven transitions of care practice model for prescribing oral antimicrobials at hospital discharge. JAMA Netw Open 2022;5:e2211331. doi: 10.1001/jamanetworkopen.2022.11331 35536577 PMC9092199

